# [Retracted] Expression of P2X7R in breast cancer tissue and the induction of apoptosis by the gene-specific shRNA in MCF-7 cells 

**DOI:** 10.3892/etm.2025.12895

**Published:** 2025-05-27

**Authors:** Chao Tan, Li Han, Lili Zou, Chunhua Luo, Aihua Liu, Xiejing Sheng, Dee Xi

Exp Ther Med 10:1472–1478, 2015; DOI: 10.3892/etm.2015.2705

Following the publication of the above paper, it was drawn to the Editor’s attention by a concerned reader that, regarding the flow cytometric plots shown in Fig. 7 on p. 936, the data panels in Fig. 7B and C appeared to show similar groupings of dots, which would not have been anticipated if these experiments had been performed discretely under different experimental conditions, suggesting a fundamental flaw either in the way in which these experiments were performed, or in how the results were outputted.

The Editor of *Experimental and Therapeutic Medicine* has decided that this paper should be retracted from the Journal on account of a lack of confidence in the presented data. The authors were asked for an explanation to account for these concerns, but the Editorial Office did not receive a reply. The Editor apologizes to the readership for any inconvenience caused.

